# The Acute Neutrophil Response Mediated by S100 Alarmins during Vaginal *Candida* Infections Is Independent of the Th17-Pathway

**DOI:** 10.1371/journal.pone.0046311

**Published:** 2012-09-25

**Authors:** Junko Yano, Jay K. Kolls, Kyle I. Happel, Floyd Wormley, Karen L. Wozniak, Paul L. Fidel

**Affiliations:** 1 Department of Microbiology, Immunology and Parasitology, Louisiana State University Health Sciences Center, New Orleans, Louisiana, United States of America; 2 Department of Genetics, Louisiana State University Health Sciences Center, New Orleans, Louisiana, United States of America; 3 Section of Pulmonary/Critical Care Medicine, Department of Medicine, Louisiana State University Health Sciences Center, New Orleans, Louisiana, United States of America; 4 Department of Biology, University of Texas San Antonio, San Antonio, Texas, United States of America; King's College London Dental Institute, United Kingdom

## Abstract

Vulvovaginal candidiasis (VVC) caused by *Candida albicans* affects a significant number of women during their reproductive ages. Clinical observations revealed that a robust vaginal polymorphonuclear neutrophil (PMN) migration occurs in susceptible women, promoting pathological inflammation without affecting fungal burden. Evidence to date in the mouse model suggests that a similar acute PMN migration into the vagina is mediated by chemotactic S100A8 and S100A9 alarmins produced by vaginal epithelial cells in response to *Candida*. Based on the putative role for the Th17 response in mucosal candidiasis as well as S100 alarmin induction, this study aimed to determine whether the Th17 pathway plays a role in the S100 alarmin-mediated acute inflammation during VVC using the experimental mouse model. For this, IL-23p19^−/−^, IL-17RA^−/−^ and IL-22^−/−^ mice were intravaginally inoculated with *Candida*, and vaginal lavage fluids were evaluated for fungal burden, PMN infiltration, the presence of S100 alarmins and inflammatory cytokines and chemokines. Compared to wild-type mice, the cytokine-deficient mice showed comparative levels of vaginal fungal burden and PMN infiltration following inoculation. Likewise, inoculated mice of all strains with substantial PMN infiltration exhibited elevated levels of vaginal S100 alarmins in both vaginal epithelia and secretions in the vaginal lumen. Finally, cytokine analyses of vaginal lavage fluid from inoculated mice revealed equivalent expression profiles irrespective of the Th17 cytokine status or PMN response. These data suggest that the vaginal S100 alarmin response to *Candida* does not require the cells or cytokines of the Th17 lineage, and therefore, the immunopathogenic inflammatory response during VVC occurs independently of the Th17-pathway.

## Introduction

Vulvovaginal candidiasis (VVC), caused by *Candida* species, is an opportunistic fungal infection that affects approximately 75% of healthy women of childbearing age [1]. While historically VVC and recurrent VVC (RVVC) have been attributed to a putative local immune deficiency, several studies using a mouse model of *Candida* vaginitis and many cross-sectional studies evaluating women with RVVC have shown that protection is not mediated by *Candida*-specific adaptive immunity [2], [3]. In contrast, results from a human live challenge study revealed that protection occurs in the absence of any inflammatory response, whereas symptomatic infection is associated with a vaginal cellular infiltrate consisting exclusively of polymorphonuclear neutrophils (PMNs) [4]. The ensuing acute inflammatory response by PMNs appears to be intimately associated with symptoms of vaginitis.

Similar to clinical observations, the presence of vaginal PMNs is also seen in the experimental mouse model of *Candida* vaginitis. We recently reported evidence implicating that the PMN migration is mediated by chemotactic S100A8 and S100A9 alarmins produced by vaginal epithelial cells in response to *Candida* [5]. The production of the S100 alarmins by vaginal epithelial cells was elevated in inoculated mice exhibiting a high PMN influx (simulating a symptomatic condition) compared to those with low PMNs (simulating an asymptomatic condition). Furthermore, PMN chemotactic activity of vaginal lavage fluid was dramatically reduced following antibody neutralization of S100A8 *in vitro*. These findings suggested that in the mouse model, epithelial cell-derived S100A8 alarmin mediates the acute PMN response that leads to the pathological inflammation associated with vaginitis.

Th17 is a recently discovered population of CD4+ effector T cells whose lineage and cytokine profile are unique from the classical Th1 and Th2 subsets [6]. Th17 cells predominantly produce IL-17 (A and F), IL-21 and IL-22. IL-23 is critical for the expansion/maintenance and the generation of a robust Th17 response. There is emerging evidence implicating that Th17 cells play a predominant role in coordinating protective mucosal immune response against oropharyngeal candidiasis (OPC) and chronic mucocutaneous candidiasis (CMC) [7], [8]. In these diseases, a Th17 response appears to provide protection by inducing neutrophil-activating factors, inflammatory chemokines and antimicrobial proteins that ameliorate the infection. In gastrointestinal candidiasis, however, an exacerbated Th17 response to *Candida* leads to uncontrolled inflammation responsible for the immunopathology of the disease [9]. Thus, despite the common pathogen, host response to *Candida* through a Th17 response has dichotomous effects depending on the site of infection. To date, the role of Th17 cells in coordinating host responses to *Candida* in the vaginal mucosa remains controversial with limited studies [10]–[12].

The production of S100 alarmins is reported to be induced in part by cytokines of the Th17 lineage and IL-17 receptor-mediated signaling within target cells, including epithelial cells [13]. IL-22 produced by Th17 cells and activated dendritic cells is also known to initiate innate immune responses in respiratory and gut epithelial cells [14]–[16]. IL-22, together with IL-17, contributes to inflammatory responses by inducing epithelial cells to produce antimicrobial peptides and chemotactic mediators such as S100 alarmins [17]. However, there is no information whether the Th17 response is involved in the S100 alarmin-mediated PMN migration during *Candida* vaginitis.

The purpose of this study was to evaluate the role of the Th17 pathway in the S100 alarmin-mediated PMN migration during *Candida* vaginitis using the experimental mouse model.

## Results

### Mice deficient in cytokines of Th17 lineage are equally susceptible to vaginal colonization with *Candida*

**Figure 1:**
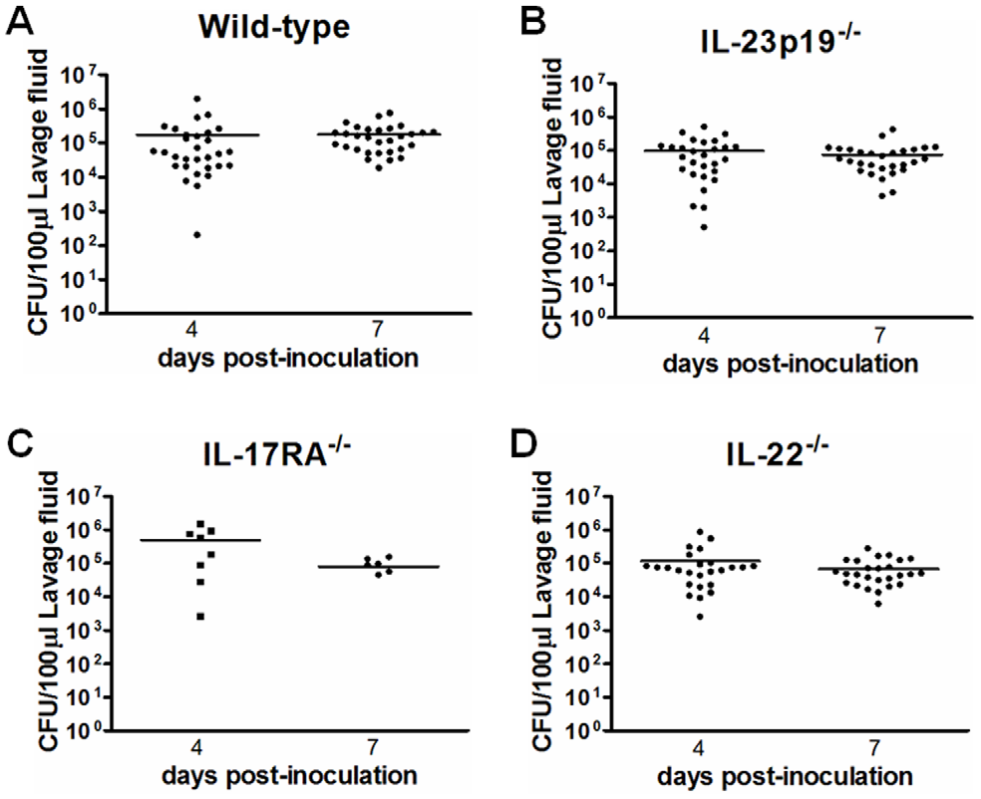
Vaginal *Candida* burden. CFU/100 µl of lavage fluids from inoculated (A) wild-type, (B) IL-23p19^−/−^, (C) IL-17RA^−/−^ and (D) IL-22^−/−^ mice was quantified on day 4 and 7 post-inoculation. Each point represents an individual mouse, and the horizontal bar indicates the geometric means. The results are cumulative from 3 separate experiments with 7 to 10 mice per group except for IL-17RA^−/−^ mice (data include 1 experiment) whose availability was limited due to difficulty in breeding.

To evaluate whether *Candida* is able to establish vaginal colonization in mice deficient in Th17 lineage, estrogenized IL-23p19^−/−^, IL-22^−/−^, IL-17RA^−/−^ and wild-type mice were inoculated with *Candida*, and vaginal lavage fluids were collected on day 4 and 7 post-inoculation and evaluated for fungal burden. All strains of mice were colonized with *Candida* and showed similar levels of vaginal fungal burden which persisted throughout the study period (**Figure 1**).

### Vaginal PMN influx occurs in Th17-deficient mice following inoculation with *Candida*

**Figure 2:**
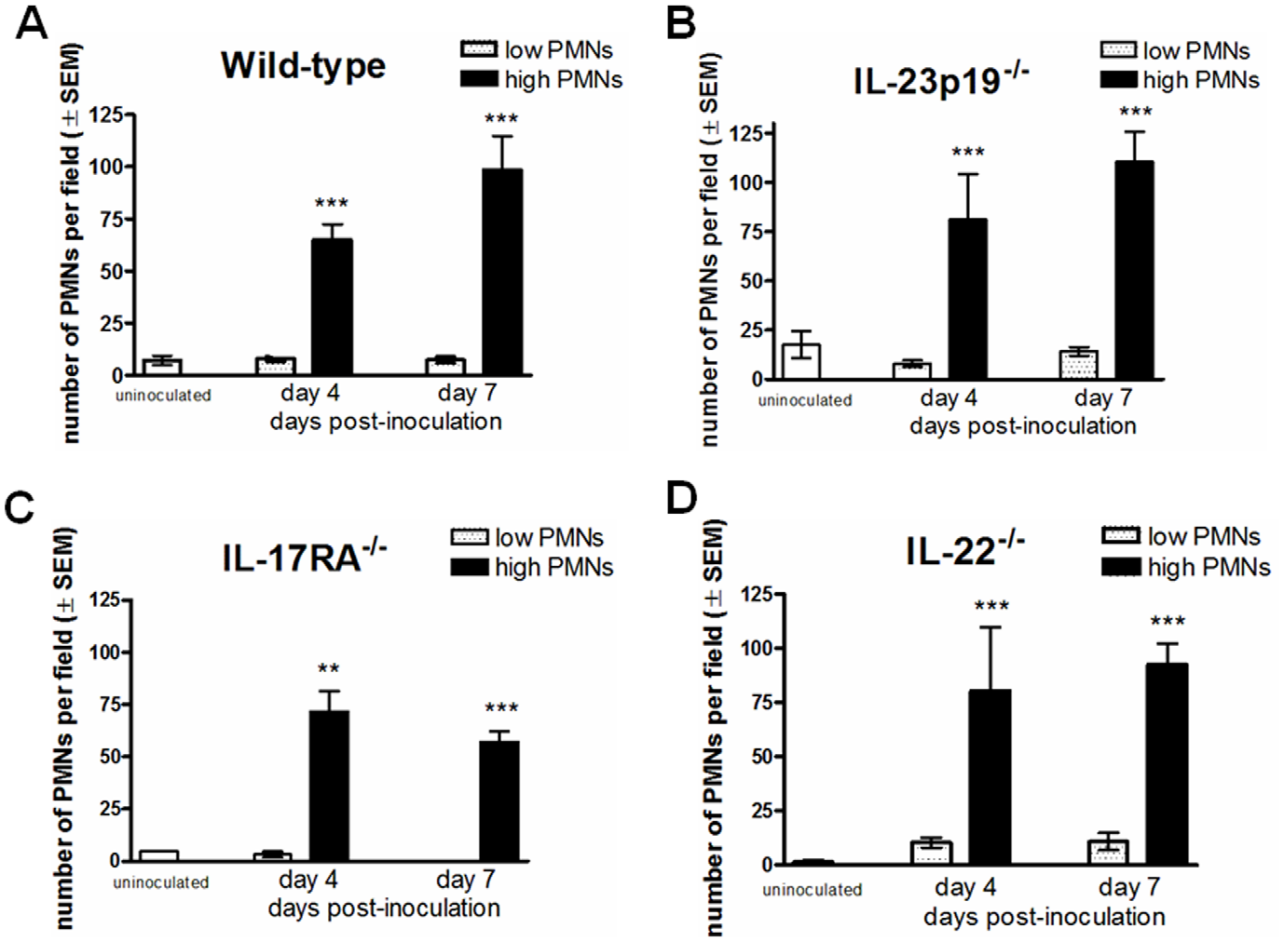
Vaginal PMNs post-inoculation. PMNs identified by pap smear preparations of vaginal lavage fluid from uninoculated or inoculated wild-type, IL-23p19^−/−^, IL-17RA^−/−^ and IL-22^−/−^ mice with high PMNs were examined at ×400 magnification and enumerated per microscopic field. High PMNs were defined as ≥50 PMNs/field; low PMNs were defined as <25 PMNs/field. The results are cumulative for 3 separate experiments (1 experiment for IL-17RA^−/−^) with 7 to 10 mice per group. Statistical analyses were performed comparing PMN values between low PMN and high PMN groups. **, p<0.01; ***, p<0.0001. SEM, standard error of the mean.

Based on clinical and animal studies showing a robust PMN infiltration into the vagina during symptomatic condition of infection [4], [5], vaginal lavage fluids from inoculated animals were also examined for the presence of vaginal PMNs. As shown in **Figure 2**, the number of vaginal PMNs was significantly increased in inoculated mice of all strains compared to uninoculated mice (p<0.05 in all strains). Furthermore, the patterns of vaginal PMN infiltration were comparable between all groups of animals irrespective of the cytokine status (p>0.05); the majority of inoculated animals showed >45 PMNs per high-powered microscopic field while uninoculated animals had very few PMNs if present. Inoculated mice were also evaluated for vaginal fungal burden and PMNs on day 10 post-inoculation and showed equivalent results to day 4 and day 7 post-inoculation (data not shown).

### Chemotactic S100 alarmins are produced by vaginal epithelium in Th17-deficient mice following interaction with *Candida*

**Figure 3:**
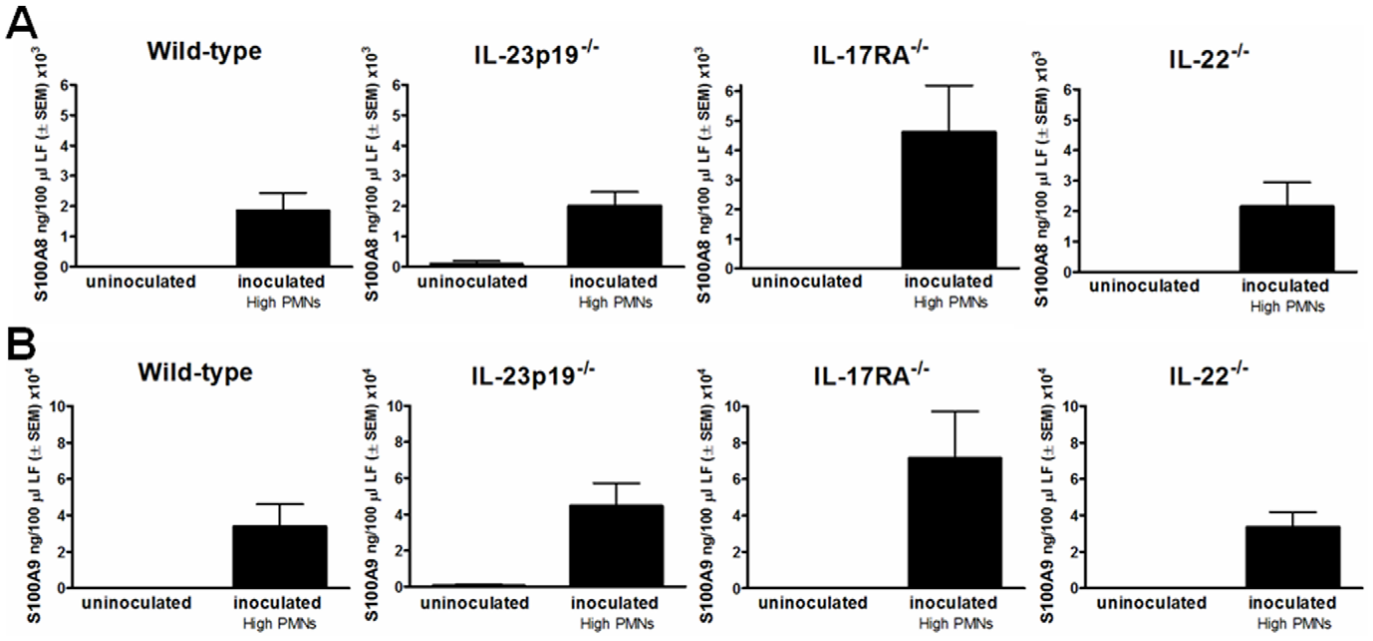
Vaginal S100A8 and S100A9. Vaginal lavage fluid from uninoculated or inoculated wild-type, IL-23p19^−/−^, IL-17RA^−/−^ and IL-22^−/−^ mice with high PMNs were evaluated for (A) S100A8 and (B) S100A9 concentrations by ELISA. The results are cumulative data of 1 to 3 repeat experiment(s) testing lavage samples collected on day 7 post-inoculation. LF, lavage fluid. SEM, standard error of the mean.

**Figure 4:**
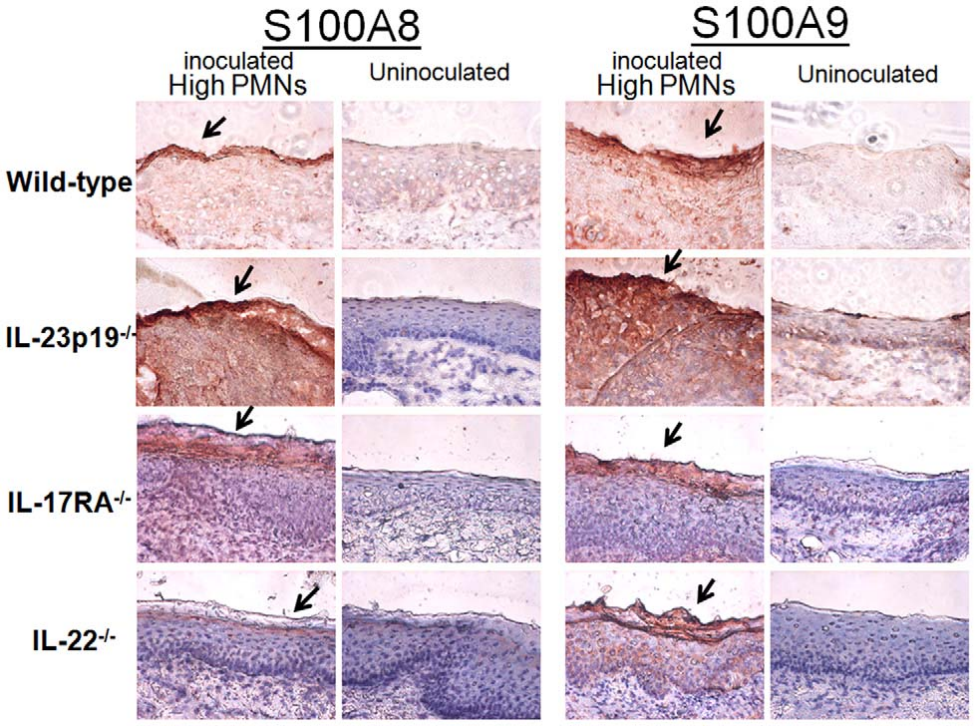
Presence of S100A8 and S100A9 on vaginal epithelium following interaction with *Candida*. Vaginal tissue sections from uninoculated or inoculated mice with high PMNs were stained with anti-S100A8 or S100A9 antibodies. Images are shown at ×400 magnification. Arrows represent epithelium positively stained for S100A8 or S100A9. Images show representative results of 15 uninoculated and 30 inoculated animals on day 7 post-inoculation.

Based on animal studies showing elevated levels of S100A8 and S100A9 alarmins in inoculated mice with high numbers of vaginal PMNs [5], lavage fluids from inoculated mice 7 days post-inoculation were evaluated for S100A8 and S100A9 by ELISA. The results in **Figure 3** show that the concentrations of both S100A8 and S100A9 were significantly increased in lavages from inoculated mice of all strains with high PMNs compared to those from uninoculated mice (P<0.05 in each strain). No significant differences were detected in the levels of S100A8 or S100A9 between the four inoculated strains of mice (P>0.05). It was also important to confirm that the S100 alarmins were produced by vaginal epithelial cells in response to *Candida* similar to our previous studies [5]. For this, vaginal tissue sections were collected from inoculated and uninoculated knockout or wild-type mice and stained for S100A8 and S100A9 by immunohistochemistry. The results in **Figure 4** show that increased levels of both proteins were present on vaginal epithelium of inoculated (day 7) mice of all strains with high PMNs compared to epithelium of uninoculated mice. In addition, the epithelial cells positively stained for the S100 alarmins were localized at the outer layer of the vaginal epithelium, exhibiting more intense staining patterns in the apical surface where cells are exposed to the vaginal lumen. Positive staining with the pan-epithelial cell markers (AE1/AE3) provided confirmation that epithelial cells were being examined in the specimens (data not shown).

### Vaginal inflammatory cytokine profile was comparable between wild-type and Th17-deficient mice and remained unaffected following inoculation irrespective of the PMN influx levels

**Figure 5:**
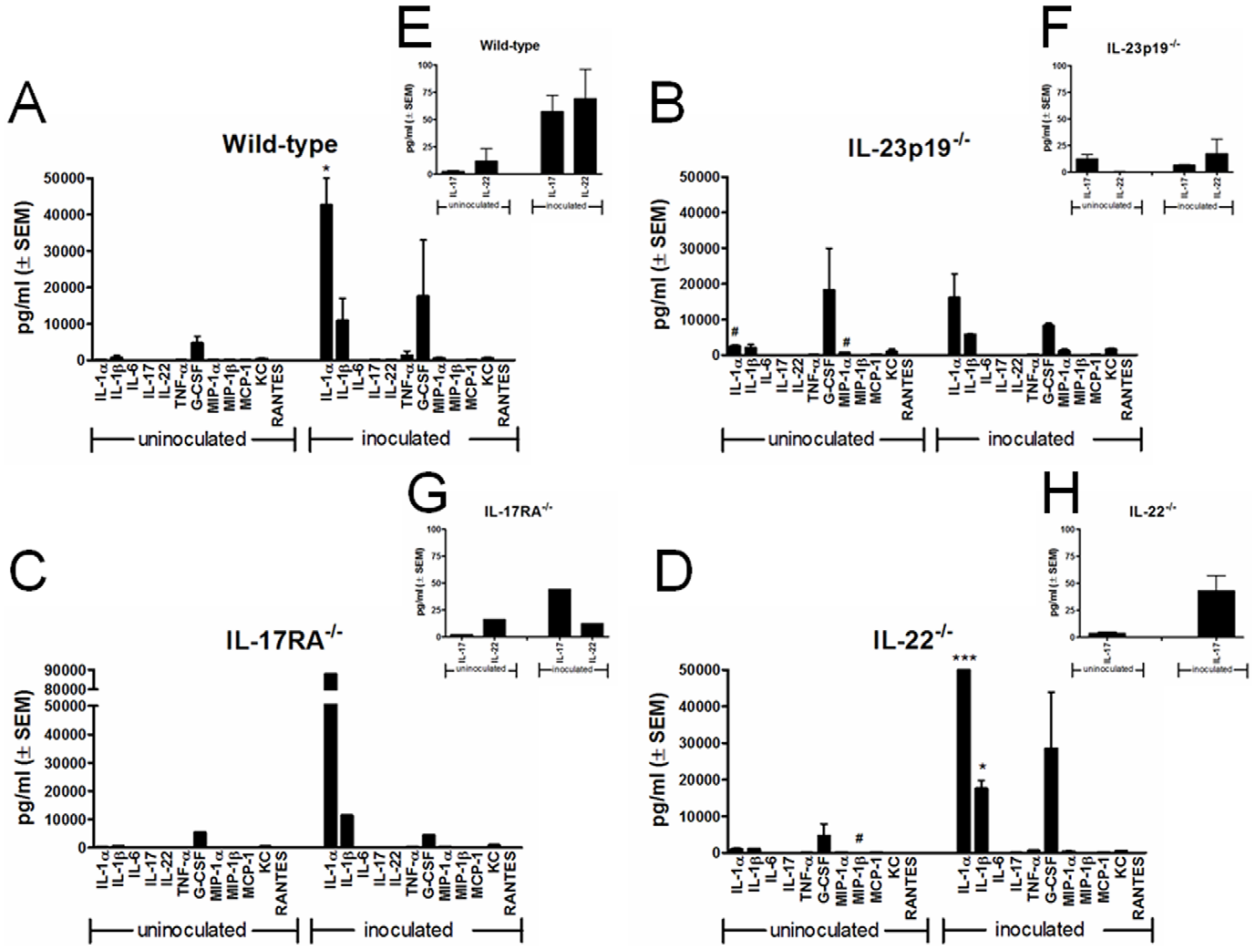
Vaginal proinflammatory cytokine and chemokine expression. Vaginal lavage fluid from uninoculated or inoculated (A) wild-type, (B) IL-23p19^−/−^, (C) IL-17RA^−/−^ and (D) IL-22^−/−^ mice with high PMNs were evaluated for the presence of inflammatory cytokines and chemokines using the Bioplex protein array system or IL-22 by ELISA. (E)–(H) indicate concentrations of IL-17 and IL-22 from the above data in a lower y-scale. The results are cumulative data using pooled lavage (mice/group) post-inoculated (day 4 and 7) from 2 separate experiments with 7 to 10 animals per group, except IL-17RA^−/−^ mice which include 1 experiment due to lack of availability of animals. *, p<0.05; ***, p<0.0001. SEM, standard error of the mean.

A series of inflammatory cytokines and chemokines were evaluated in lavage fluids to 1) confirm the lack of associated Th17 cytokines in Th17-compromised mice and 2) reveal any other cytokines that may be contributing to the symptomatic condition. Pro-inflammatory cytokines included IL-1α, IL-1β, IL-17, IL-22, TNF-α and G-CSF. Chemokines included MIP-1α, MIP-1β, MCP-1, KC and RANTES. As shown in **Figure 5 A–D**, levels of both pro-inflammatory cytokines and chemokines were comparable among all strains of mice and showed minimal changes locally following inoculation. The exception was an increase in IL-1α levels in wild-type and IL-22^−/−^ mice (p<0.05 and p<0.0001, respectively) and IL-1β in IL-22^−/−^ mice (p<0.05) following inoculation. When analyzed comparatively between lavages from mice with high PMNs and low PMNs, the cytokine and chemokine levels remained unaffected irrespective of the PMN status (data not shown). In addition to Th17 cells being the major source of IL-17 and IL-22 in various inflammatory conditions, other innate immune cells, such as γδ T cells and innate lymphocytic cells (ILCs), are also known to secrete appreciable amounts of these cytokines and contribute to inflammatory diseases [18]–[20]. For this reason, it was important to additionally evaluate the presence of IL-17 and IL-22 in vaginal lavage fluid from inoculated wild-type and Th17-compromised animals. Results showed that wild-type mice exhibited a moderate elevation in IL-17 and IL-22 following inoculation although this did not reach statistical significance (p>0.05 for both cytokines) (**Figure 5E**), whereas IL-23p19^−/−^ mice failed to produce IL-17 and IL-22 in response to *Candida* (**Figure 5F**). IL-17RA^−/−^ and IL-22^−/−^ mice showed a small increase in IL-17 following inoculation but this was not statistically significant (p>0.05) (**Figure 5G and H**).

## Discussion

Despite the strong evidence of Th17 cells modulating host responses to various forms of candidiasis [7]–[9], no role of the Th17 pathway of immunity could be demonstrated at any level for fungal burden, PMN migration and S100 alarmin production in the vagina during experimental vaginal candidiasis. All strains of mice deficient in cytokines of Th17-lineage were equally susceptible to vaginal colonization with *Candida* compared to wild-type mice. Likewise, all animals were able to elicit a robust vaginal PMN response and S100 induction/secretion by vaginal epithelium following inoculation. Although these findings were unexpected based on the strong role of the Th17 response in PMN migration and induction of S100 alarmins, the lack of roles for the Th17 pathway may be explained two-fold. First, previous studies showed that vaginal epithelial cells are a primary source of S100 alarmins following vaginal inoculation with *Candida* where early *Candida* adherence to vaginal epithelium within the first 24 h is a critical event in initiating the PMN response [5]. Thus, the S100 alarmin induction during vaginal infection is mediated exclusively by a direct interaction between epithelial cells and *Candida*. Our current data extends this to exclude any role for Th17 in the process. Second, while host responses by CD4^+^ T cells are generally required for protection against mucosal candidiasis, no role has been shown for either local or systemic CD4^+^ T cells against VVC [2], [3]. The lack of a protective role for CD4^+^ T cells is further supported by accumulating evidence of immunomodulatory mechanisms towards adaptive responses [21]–[23]. Hence, it may not be surprising that this also includes Th17-type responses as well as Th22 cells. Of note, there is the possibility that IL-17 and IL-22 are produced by other cellular sources. These may include γδ T cells and other innate lymphoid cells in the vaginal mucosa that may act on epithelial cells and contribute to the induction of S100 alarmin-mediated inflammation during vaginal infection. However, while IL-17 and IL-22 were increased moderately in wild-type mice following inoculation, both cytokines were virtually negligible in IL-23p19^−/−^ mice, suggesting that the cytokines by these other cell sources is minimal at best.

Despite strong evidence shown here against a role for Th17 cells and the fact that Th17 cells are known to be the major producer of IL-17 and IL-22, we recognize that IL-22 alone still may be a driving force in the PMN response and that IL-22 derived from innate immune cells (e.g. activated dendritic cells) could initiate the S100 response in vaginal epithelial cells. However, our results from IL-22^−/−^ mice showing elevated vaginal S100 alarmins and PMN infiltration in response to *Candida* at equivalent levels to wild-type mice reduces the possibility of IL-22 being a primary cytokine in the S100 response. Furthermore, the epithelial cell S100 alarmin response occurs concomitantly with the exclusive presence of PMNs within 48 h post-inoculation. Thus, it is unlikely that contributions of S100 alarmins are made by other nonresident innate or adaptive cells. However, we recognize that other resident innate cells could contribute at low levels. Once the S100 alarmins and PMNs are present, the inflammatory process is in place and continues for a considerable time (for up to 30 days post-inoculation in previous studies evaluating mice longitudinally [5]). Hence, the role for the Th17 pathway in this model would likely be evident early as much as later in the infection. Taken together, we hypothesize that the vaginal S100 induction and accompanied PMN response are initiated via an innate pathogen recognition mechanism by vaginal epithelium and occurs independently of the cells or cytokines of the Th17 lineage.

Of Note, there are a few cases of contrary data that challenge this hypothesis. One is a clinical study reporting that PBMCs from dectin-1^−/−^ women exhibited impaired IL-6 and IL-17 production and in turn, increased susceptibility to familial chronic mucocutaneous candidiasis and RVVC [10]. However, the results are from a small number of patients that interestingly did not suffer additionally with OPC that has a strong correlate to Th17 deficiency [7]. Thus, the incidence of RVVC could have been unrelated to the dectin-1 deficiency and simply coincidental. In addition, another clinical study reported that IL-6, IL-12 and IL-23 were only detected in a small percentage of RVVC women, suggesting a lack of a role for either Th1- or Th17-associated immunity [11]. These inconsistent clinical observations implicate the need for additional studies to properly examine host immunological factors associated with RVVC and any formal role of the Th-17 cytokines to resistance. Another study evaluating a role of the Th17 pathway in VVC included an animal model in which mice treated with halofuginone, an inhibitor of Th17 differentiation, produced significantly less IL-17 by vaginal CD4^+^ T cells and resulted in exacerbated vaginal infection due to impaired production of antimicrobial peptides β-defensin (BD)-2 and BD-3 by vaginal epithelial cells [12]. However, the efficacy of halofuginone for abrogation of vaginal Th17 cells is unknown in this study, and data only showed weak IL-17-mediated protection against vaginal infection or in the BD-2 response. In contrast, the present study using a more stringent system of Th17 cytokine-deficient animals clearly demonstrated a lack of roles for the Th17 pathway in both vaginal fungal burden and immunopathology associated with *Candida* vaginitis.

To provide further confirmation to the lack of participation by the Th17 pathway during infection, vaginal lavage fluids from IL-23p19^−/−^ (Th17-compromised), IL-17RA^−/−^, IL-22^−/−^ and wild-type inoculated mice were evaluated for a series of proinflammatory cytokines and chemokines. Of note, only trace amounts (ranging from 0 to 103 pg/ml) of IL-17 and/or IL-22 were detected in the wild-type and IL-23p19^−/−^ mice under both uninoculated and inoculated conditions, indicating a lack of Th17-associated cytokine responses following vaginal *Candida* challenge. The lack of IL-17/IL-22 cytokines was also confirmed when comparing data between inoculated animals with high PMNs (symptomatic condition) and low PMNs (asymptomatic condition) (data not shown). These data provide yet additional evidence against a link of the cytokines to resistance to RVVC in the dectin-1^−/−^ clinical case. Furthermore, no proinflammatory cytokines and chemokines showed a remarkable increase in response to *Candida in vivo*. This finding is consistent with previous studies that reported the absence of adaptive immune cytokines during vaginitis in humans and mice [24]–[26]. The only significant increase detected following inoculation was IL-1α in the wild-type and IL-22^−/−^ mice, and IL-1β in IL-22^−/−^ mice, with trends toward similar increases in the other strains. IL-1α is known to be produced constitutively by epithelial cells whereas IL-1β is predominantly produced by activated phagocytes [27], [28]. The upregulation of IL-1α and IL-1β in this study could be considered as a consequence of inflammation and cell damage and a process to restore the epithelial barrier integrity following the establishment of the disease. Of note, Wagner *et al.* recently reported that expression of IL-1α and IL-1β was unaffected in vaginal epithelial cells challenged with *C. albicans in vitro* [29]. Thus, it is unlikely that IL-1α and IL-1β would have a direct effect on the initiation of PMN migration in response to *Candida*. This is consistent with virtually identical results observed for all parameters evaluated (CFUs, PMNs, and local S100 concentrations) irrespective of the local presence of IL-1α or IL-1β in the microenvironment. Signaling through the IL-1 receptor (IL-1R), on the other hand, has been shown to induce expression of S100A8 and S100A9 in oral epithelial cells following bacterial invasion [30]. It would be interesting to investigate whether IL-1 and IL-1R signaling contribute to the production of the S100 alarmins by vaginal epithelial cells as a resistance mechanism, but ultimately help contribute to the exacerbation of disease.

Studies to dissect immune mechanisms against mucosal candidiasis have revealed the divergence of tissue-specific host responses against *Candida*. Within the view of Th1/Th2 paradigm, Th1 responses were believed to mediate resistance against certain infections with *Candida* such as OPC by promoting protection primarily via interferon (IFN)-γ [31]. Since the discovery of the Th17-axis of immunity, there is accumulating evidence for protective roles of Th17-axis against OPC and CMC where ensuing IL-17 and IL-22 signal epithelial cells to produce PMN-recruiting chemokines and other soluble factors such as S100 proteins [7], [13], [14], opposed to the GI tract where the activation of Th17 cells in response to *Candida* has been implicated to induce pathological inflammation [9]. Here, we provide additional data in an experimental animal model showing no role either way for the Th17-type responses in immunopathology of vaginal candidiasis. The evidence from these studies extends the understanding that susceptibility and resistance to symptomatic VVC occurs in the absence of adaptive immune responses and likely to be mediated solely by innate immunity through the direct interaction of *Candida* with the vaginal epithelium.

## Materials and Methods

### Ethics Statement

This study was carried out in accordance with the recommendations in the Guide for the Care and Use of Laboratory Animals of the National Institutes of Health. Mice were housed at LSU Health Sciences Center Animal Care facility. All animal protocols were reviewed and approved by the Institutional Animal Care and Use Committee (IACUC) of the LSU Health Sciences Center. All efforts were made to minimize pain and discomfort in the animals.

### Mice

Breeders deficient in IL-23p19 and IL-22 were obtained from Dr. Kyle Happel and Dr. Jay Kolls, respectively (LSU Health Sciences Center, New Orleans, LA). Breeders deficient in IL-17RA were obtained from Taconic Farms, Hudson, NY. Females 6 to 10 weeks of age from the breeding colonies above were used throughout the studies. Age-matched female C57BL/6 mice were purchased from Charles River at the National Cancer Institute, Frederick, MD and tested in parallel as wild-type controls.

### Vaginal *Candida* inoculation

Vaginal inoculation with *Candida* was conducted as previously described [3], [32]. Briefly, mice were administered 0.1 mg of β-estradiol 17-valerate (Sigma Chemical Co., St. Louis, MO) in 100 µl of sesame oil (Sigma) by subcutaneous injection 72 h prior to inoculation and then weekly until the completion of the study period. Estrogen-treated (estrogenized) mice were intravaginally inoculated by introducing 20 µl of phosphate-buffered saline (PBS) containing 5×10^4^ *C. albicans* blastoconidia from a stationary-phase culture (12 to 18 h of culture at 25°C in phytone-peptone broth with 0.1% glucose) into the vaginal lumen. Uninoculated control mice were estrogenized and intravaginally challenged with sterile PBS. Separate groups of 7 to 10 mice were evaluated on 4, 7 and 10 days post-inoculation. On euthanized animals, vaginal lavages were performed using 100 µl of sterile PBS with gentle aspiration and agitation for 30 s. To assess vaginal fungal burden, the lavage fluid was cultured at 1∶10 serial dilutions on Sabourand-dextrose agar plates (BD Diagnostics, Sparks, MD) supplemented with gentamicin (Invitrogen, Carlsbad, CA), and CFUs were enumerated after incubation at 34°C for 48 h. Supernatants of the lavage fluid were sterile filtered and stored at −70°C until use.

### Quantification of vaginal PMNs

Smears of 10 µl vaginal lavage fluids from each inoculated or uninoculated mouse were prepared on glass slides and stained by the standard Papanicolaou technique (pap smear). When infiltrating leukocytes were present, PMNs were identified to be the predominant cell type by the trilobed nuclear morphology. Vaginal PMNs were enumerated in 5 nonadjacent high-powered fields (400×) per mouse by light microscopy and averaged.

### ELISA

The amount of S100A8 and S100A9 in vaginal lavage fluids from inoculated and uninoculated mice were quantified by a standard enzyme-linked immunosorbent assay (ELISA) as previously described [5]. Briefly, 96-well EIA/RIA plates (Costar, Corning, NY) were coated with monoclonal rat anti-mouse S100A8 or S100A9 antibodies (2 µg/ml; R&D Systems, Minneapolis, MN) overnight at 4°C. The plates were blocked for 1 h at 37°C and washed with ELISA wash buffer (0.5% Tween 20 in PBS). Standards (serially diluted recombinant mouse S100A8 or S100A9; R&D Systems) and lavage fluid supernatants (at dilutions ranging from 1∶10 to 1∶10^5^) were added in triplicate and incubated for 2 h at 37°C. Following washing, the plates were incubated with primary antibodies (1 µg/ml; polyclonal goat anti-mouse S100A8 or S100A9) for 1 h at 37°C, washed 5 times and then incubated with a secondary antibody (biotinylated anti-goat IgG, 0.05 µg/ml) for 1 h at 37°C. After washing, the plates were incubated with streptavidin-horseradish peroxidase (Bio-Rad) for 30 min at room temperature, washed 5 times and reacted with one-step ultra tetramethylbenzidine (TMB; Thermo Fisher Scientific, Rockford, IL). The colorimetric reaction was stopped with sulfuric acid (2 N) when it reached the optimal intensity. The absorbance was read at 450 nm on a Multiskan Ascent microplate photometer (Labsystems, Helsinki, Finland). The results were expressed as nanograms per 100 µl of lavage fluid.

### Immunohistochemistry

Levels of S100A8 and S100A9 expression by vaginal epithelia were examined by immunohistochemistry as previously described [5]. Briefly, vaginae from inoculated and uninoculated mice were excised and embedded in Tissue-Tek cryomolds (Miles Corp., Elkhart, ID) containing optimum cutting temperature (OCT) medium (Sakura Finetek USA, Torrance, CA) in an orientation that allowed cross-sectional cutting. Tissue blocks were frozen at −70°C and sectioned in 6 µm. Tissues were collected on glass slides, fix in ice-cold acetone for 5 min and stored at −20°C until use. Following hydration, tissues were stained using the cell and tissue staining kit HRP-3-amino-9-ethylcarbazole (AEC; R&D Systems) with appropriate primary antibodies. Briefly, tissues were treated with peroxidase, goat serum, avidin and biotin blocking buffers and then incubated with monoclonal rat anti-mouse S100A8 or S100A9 antibody (10 µg/ml; R&D Systems), monoclonal mouse anti-human AE1/AE3 antibody (epithelial cytokeratin markers, 5 µg/ml; MP Biomedicals, Solon, OH), or isotype controls (rat IgG2a, rat IgG2b and mouse IgG1) overnight at 4°C. The slides were washed and incubated with biotinylated anti-rat IgG antibodies (R&D Systems) or anti-mouse IgG F(ab′)_2_ fragments (Thermo) for 1 h at room temperature. The slides were then washed and incubated with streptavidin-HRP for 30 min. Finally, the slides were washed and reacted with AEC chromagen. The tissues were counterstained with CAT hematoxylin (Biocare Medical, Concord, CA), preserved in aqueous mounting medium (R&D Systems) and observed at 400× magnification by light microscopy.

### Cytokine analysis

Vaginal lavage fluids from inoculated and uninoculated mice were analyzed for a series of inflammatory cytokines and chemokines using the Bio-Plex Array System (Luminex; Bio-Rad). Vaginal lavage supernatants were assayed for the presence of IL-1α, IL-1β, IL-6, IL-17, granulocyte-colony stimulating factor (G-CSF), keratinocyte-derived chemokine (KC), macrophage chemoattractant protein (MCP)-1, macrophage inflammatory protein (MIP)-1α, MIP-1β, regulated upon activation normal T cell expressed and secreted (RANTES) and tumor necrosis factor (TNF)-α. The fluorescence was measured on a Bio-Plex 200 system array reader (Bio-Rad). Levels of IL-22 were separately measured by the standard ELISA (R&D Systems). The absorbance was read at 450 nm on a microplate reader. The results were expressed as picograms per 100 µl of lavage fluid.

### Statistics

The unpaired Student's *t* test and ANOVA were used to analyze data. Significant differences were defined at a confidence level where *P* was <0.05 and evaluated using GraphPad Prism version 4 (GraphPad Software, San Diego, CA).

## References

[1] Sobel JD, Faro S, Force RW, Foxman B, Ledger WJ, et al (1998) Vulvovaginal candidiasis: Epidemiologic, diagnostic, and therapeutic considerations. Am J Obstet Gynecol 178: 203–211. 9500475 10.1016/s0002-9378(98)80001-x

[2] Fidel PL Jr, Sobel JD (1996) Immunopathogenesis of recurrent vulvovaginal candidiasis. Clin Microbiol Rev 9: 335–348. 8809464 10.1128/cmr.9.3.335 PMC172897

[3] Fidel PL Jr, Sobel JD (1999) Murine models of *Candida* vaginal infections, p. 741–748. In: Zak O, Sande M. Experimental models in antimicrobial chemotherapy, 2nd ed. London UK: Academic Press Ltd. pp.741–748.

[4] Fidel PL Jr, Barousse M, Espinosa T, Ficarra M, Sturtevant J, et al (2004) An intravaginal live *Candida* challenge in humans leads to new hypothesis for the immunopathogenesis of vulvovaginal candidiasis. Infect Immun 72: 2939–2946. 15102806 10.1128/IAI.72.5.2939-2946.2004 PMC387876

[5] Yano J, Lilly E, Barousse M, Fidel PL Jr (2010) Epithelial cell-derived S100 calcium-binding proteins as key mediators in the hallmark acute neutrophil response during *Candida* vaginitis. Infect Immun 78: 5126–5137. 20823201 10.1128/IAI.00388-10 PMC2981313

[6] Onishi RM, Gaffen SL (2010) Interleukin-17 and its target genes: mechanisms of interleukin-17 function in disease. Immunology 129: 311–321. 20409152 10.1111/j.1365-2567.2009.03240.x PMC2826676

[7] Conti HR, Shen F, Nayyar N, Stocum E, Sun JN, et al (2009) Th17 cells and IL-17 receptor signaling are essential for mucosal host defense against oral candidiasis. J Exp Med 206: 299–311. 19204111 10.1084/jem.20081463 PMC2646568

[8] Eyerich K, Foerster S, Rombold S, Seidl HP, Behrendt H, et al (2008) Patients with chronic mucocutaneous candidiasis exhibit reduced production of Th17-associated cytokines IL-17 and IL-22. J Invest Dermatol 128: 2640–2645. 18615114 10.1038/jid.2008.139

[9] Zelante T, De Luca A, Bonifazi P, Montagnoli C, Bozza S, et al (2007) IL-23 and the Th17 pathway promote inflammation and impair antifungal immune resistance. Eur J Immunol 37: 2695–2706. 17899546 10.1002/eji.200737409

[10] Ferwerda B, Ferwerda G, Plantinga TS, Willment JA, van Spriel AB, et al (2009) Human dectin-1 deficiency and mucocutaneous fungal infections. N Engl J Med 361: 1760–67. 19864674 10.1056/NEJMoa0901053 PMC2773015

[11] Lev-Sagie A, Nyirjesy P, Tarangelo N, Bongiovanni AM, Bayer C, et al (2009) Hyaluronan in vaginal secretions: association with recurrent vulvovaginal candidiasis. Am J Obstet Gynecol 201: 206.e1–206.e5. 19646572 10.1016/j.ajog.2009.05.010

[12] Pietrella D, Rachini A, Pines M, Pandey N, Mosci P, et al (2011) Th17 cells and IL-17 in protective immunity to vaginal candidiasis. PLoS One 6: e22770. 21818387 10.1371/journal.pone.0022770 PMC3144947

[13] Khader SA, Gaffen SL, Kolls JK (2009) Th17 cells at the crossroads of innate and adaptive immunity against infectious diseases at the mucosa. Mucosal Immunol 2: 403–411. 19587639 10.1038/mi.2009.100 PMC2811522

[14] De Luca A, Zalente T, D'Angelo C, Zagarella S, Fallarino F, et al (2010) IL-22 defines a novel immune pathway of antifungal resistance. Mucosal Immunol 3: 361–373. 20445503 10.1038/mi.2010.22

[15] Godinez I, Haneda T, Raffatellu M, George MD, Paixão TA, et al (2008) T cells help to amplify inflammatory responses induced by Salmonella enterica serotype Typhimurium in the intestinal mucosa. Infect Immun 76: 2008–2017. 18347048 10.1128/IAI.01691-07 PMC2346712

[16] Happel KI, Zheng M, Young E, Quinton LJ, Lockhart E, et al (2003) Cutting Edge: roles of Toll-like receptor 4 and IL-23 and IL-17 expression in response to Klebsiella pneumoniae infection. J Immunol 170: 4432–4436. 12707317 10.4049/jimmunol.170.9.4432 PMC2841978

[17] Liang SC, Tan XY, Luxenberg DP, Karim R, Dunussi-Joannopoulos K, et al (2006) Interleukin (IL)-22 and IL-17 are coexpressed by Th17 and cooperatively enhance expression of antimicrobial peptides. J Exp Med 203: 2271–2279. 16982811 10.1084/jem.20061308 PMC2118116

[18] Sanos SL, Vonarbourg C, Mortha A, Diefenbach A (2011) Control of epithelial cell function by interleukin-22-producing RORgt^+^ innate lymphoid cells. Immunology 132: 453–465. 21391996 10.1111/j.1365-2567.2011.03410.x PMC3075499

[19] Rubino SJ, Geddes K, Girardin SE (2012) Innate IL-17 and IL-22 responses to enteric bacterial pathogens. Trends Immunol 33: 112–118. 22342740 10.1016/j.it.2012.01.003

[20] Ness-Schwickerath KJ, Morita CT (2011) Regulation and funtion of IL-17A- and IL-22-producing gd T cells. Cell Mol Life Sci 68: 2371–2390. 21573786 10.1007/s00018-011-0700-z PMC3152582

[21] Fidel PL Jr, Barousse M, Lounev V, Espinosa T, Chesson, et al (2003) Local immune responsiveness following intravaginal challenge with *Candida* antigen in adult women at different stages of the menstrual cycle. Med Mycol 41: 97–109. 12964841 10.1080/mmy.41.2.97.109

[22] Fidel PL Jr, Wormley FL Jr, Chaiban J, Chesson RR, Lounev V (2001) Analysis of the CD4 protein on human vaginal CD4^+^ T cells. Am J Reprod Immunol 45: 200–204. 11327546 10.1111/j.8755-8920.2001.450402.x

[23] LeBlanc DM, Barousse MM, Fidel PL Jr (2006) A role for dendritic cells in immunoregulation during experimental vaginal candidiasis. Infect Immun 74: 3213–3221. 16714548 10.1128/IAI.01824-05 PMC1479243

[24] Barousse M, Van Der Pol BJ, Fortenberry D, Orr D, Fidel PL Jr (2004) Vaginal yeast colonization, prevalence of vaginitis, and associated local immunity in adolescents. Sex Trans Infect 80: 48–53. 10.1136/sti.2002.003855 PMC1758371 14755036

[25] Saavedra M, Taylor B, Lukacs N, Fidel PL Jr (1999) Local production of chemokines during experimental vaginal candidiasis. Infect Immun 67: 5820–5826. 10531235 10.1128/iai.67.11.5820-5826.1999 PMC96961

[26] Taylor BN, Saavedra M, Fidel PL Jr (2000) Local Th1/Th2 cytokine production during experimental vaginal candidiasis. Med Mycol 38: 419–431. 11204879 10.1080/mmy.38.6.419.431

[27] Eller MS, Yaar M, Ostrom K, Harkness DD, Gilchrest BA (1995) A role for interleukin-1 in epidermal differentiation: regulation by expression of functional versus decoy receptors. J Cell Sci 108: 2741–2746. 7593315 10.1242/jcs.108.8.2741

[28] Murphy JE, Robert C, Kupper TS (2000) Interleukin-1 and cutaneous inflammation: a crucial link between innate and acquired immunity. J Invest Dermatol 114: 602–608. 10692124 10.1046/j.1523-1747.2000.00917.x

[29] Wagner RD, Johnson SJ (2012) Probiotic lactobacillus and estrogen effects on vaginal epithelial gene expression responses to *Candida albicans* . J Biomed Sci 19: 58. 22715972 10.1186/1423-0127-19-58 PMC3404894

[30] Sorenson BS, Khammanivong A, Guenther BD, Ross KF, Herzberg MC (2012) IL-1 receptor regulates S100A8/A9-dependent keratinocyte resistance to bacterial invasion. Mucosal Immunol 5: 66–75. 22031183 10.1038/mi.2011.48 PMC3476723

[31] Dongari-Bagtzoglou A, Fidel PL Jr (2005) The host cytokine responses and protective immunity in oropharyngeal candidiasis. J Dent Res 84: 966–977. 16246925 10.1177/154405910508401101

[32] Fidel PL Jr, Lynch ME, Sobel JD (1993) *Candida*-specific cell-mediated immunity is demonstrable in mice with experimental vaginal candidiasis. Infect Immun 61: 1990–1995. 8097493 10.1128/iai.61.5.1990-1995.1993 PMC280793

